# Supplementary figures and images for: Data-specific substitution models improve protein-based phylogenetics
Source: PeerJ. 2023 Aug 8;11:e15716. doi: 10.7717/peerj.15716 (PMC10416777; doi:10.7717/peerj.15716)

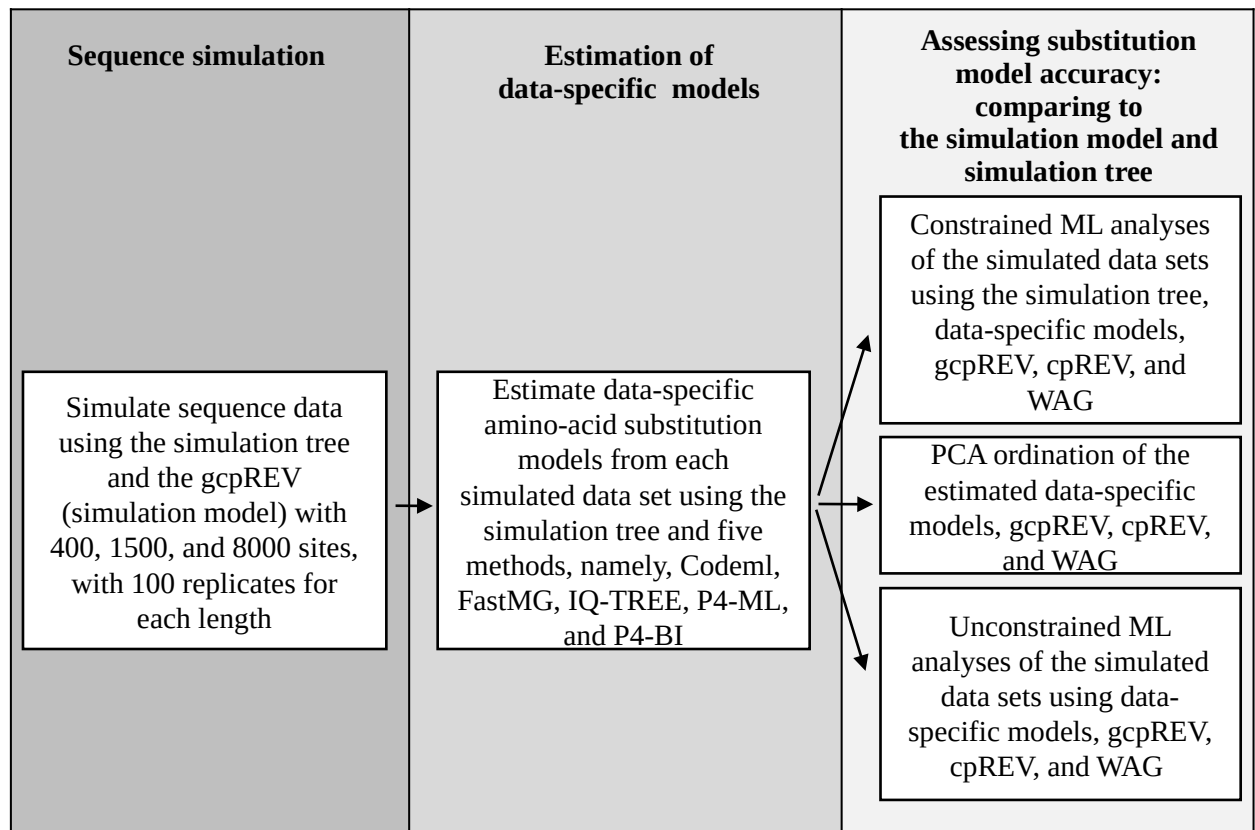

Supplement: Supplemental Information 4 — Data sets were simulated using a simulation tree and substitution model. Data-specific models were then estimated from the data sets using five methods (Codeml, IQ-TREE, FastMG, P4-ML, and P4-BI) and compared to the simulation model using ordination. Phylogenetic analyses of the data-specific models using constrained and unconstrained tree topologies were also compared to the simulation trees and model. [file peerj-11-15716-s004.pdf]
