# Supplementary material for: Data-specific substitution models improve protein-based phylogenetics
Source: PeerJ. 2023 Aug 8;11:e15716. doi: 10.7717/peerj.15716 (PMC10416777; doi:10.7717/peerj.15716)
Supplement: Supplementary file 5 [file peerj-11-15716-s001.odt]

| Study | Sequence data type | Main taxonomic groups | Taxa | Sites | Amino-acid substitution Model | Likelihood tree scores (log likelihood units) | ML tree length (substitutions per site) |
| --- | --- | --- | --- | --- | --- | --- | --- |
| Timme et al., 2012 | Nuclear | Streptophyta | 14 | 56,274 | LG+Γ4+Femp | -722,437* | 4.6 |
| Zeng et al., 2014 | Nuclear | Angiosperms | 31 | 25,589 | JTT+Γ4+I+Femp | -764,724* | 8.3 |
| Leliaert et al., 2016 | Chloroplast | Viridiplantae | 59 | 13,730 | cpREV+Γ4+Femp | -486,799* | 13.5* |
| Feuda et al., 2017 (Chang data set et al., 2015) | Nuclear | Metazoa | 77 | 51,940 | Branch-linked partition model (47 partitions) + best-fitting models | -2,321,478 | 16.6 |
| Munro et al., 2018 | Nuclear | Siphonophores | 41 | 365,699 | JTT+R7+Femp | -8,113,695 | 8.4 |
| Schulz et al., 2018 | Virus | Giant soil viruses | 128 | 5,095 | LG+R6+Femp | -472,305 | 93.0 |
| Schwentner et al., 2017 (Matrix 1) | Nuclear | Arthropods | 40 | 20,227 | LG4X | -444,511 | 6.3 |
| Toussaint et al., 2018 (DT369) | Nuclear | Papilionoidea butterflies | 138 | 52,287 | Best-fit model +R4+Femp | -630,709 | 1.8* |
| Branch-unlinked partition model (366 partitions) + Best-fitting models | -624,687 | 2.4* |
| Branch-unlinked partition model (27 partitions) + Best-fitting models | -627,253 | 1.8* |
| Irisarri et al., 2020 | Mitochondrial | Mollusca | 34 | 3,653 | MtZOA +Γ4+I+Femp | -115,156 | 19.5 |
| Koenen et al., 2020 (Chloroplast) | Chloroplast | Leguminosae | 157 | 25,094 | LG4X | -571,563* | 6.9 |
