# Supplementary material for: Data-specific substitution models improve protein-based phylogenetics
Source: PeerJ. 2023 Aug 8;11:e15716. doi: 10.7717/peerj.15716 (PMC10416777; doi:10.7717/peerj.15716)
Supplement: Supplementary file 6 [file peerj-11-15716-s002.odt]

| Amino-acid substitution models  (+Γ4+Fmod) | Non-simulation trees recovered (N of 100 replicates) | RF topological distances (mean of 100 replicates) |
| --- | --- | --- |
| gcpREV | 22 | 0.44 |
| cpREV | 24 | 0.48 |
| WAG | 25 | 0.54 |
| Codeml-estimated models | 22 | 0.44 |
| FastMG-estimated models | 19 | 0.38 |
| IQ-TREE-estimated models | 20 | 0.40 |
| P4-ML-estimated models | 20 | 0.40 |
| P4-BI-estimated models | 20 | 0.42 |
