# Supplementary material for: Data-specific substitution models improve protein-based phylogenetics
Source: PeerJ. 2023 Aug 8;11:e15716. doi: 10.7717/peerj.15716 (PMC10416777; doi:10.7717/peerj.15716)
Supplement: Supplementary file 7 [file peerj-11-15716-s003.odt]

| Software | Simulated alignments |
| --- | --- |
| 400 sites | 1500 sites | 8000 sites |
| Codeml | 14 minutes | 65 minutes | 265 minutes |
| FastMG | <1 minute | <1 minute | 3 minutes |
| IQ-TREE | 18 minutes | 45 minutes | 155 minutes |
| P4-ML | 32 minutes | 121 minutes | 576 minutes (9.6 hours) |
| P4-BI | 34 hours | 68 hours | 322 hours |
